# Segment-Specific Kinetics of mRNA, cRNA, and vRNA Accumulation during Influenza Virus Infection

**DOI:** 10.1128/JVI.02102-20

**Published:** 2021-04-26

**Authors:** Thu Phan, Elizabeth J. Fay, Zion Lee, Stephanie Aron, Wei-Shou Hu, Ryan A. Langlois

**Affiliations:** a Department of Chemical Engineering and Materials Sciences, University of Minnesota, Minneapolis, Minnesota, USA; b Biochemistry, Molecular Biology and Biophysics Graduate Program, University of Minnesota, Minneapolis Minnesota, USA; c Department of Microbiology and Immunology, University of Minnesota, Minneapolis Minnesota, USA; Cornell University

**Keywords:** influenza virus

## Abstract

Influenza A virus (IAV) is a segmented negative-sense RNA virus and is the cause of major epidemics and pandemics. The replication of IAV is complex, involving the production of three distinct RNA species, namely mRNA, cRNA, and viral RNA (vRNA), for all eight genome segments. While understanding IAV replication kinetics is important for drug development and improving vaccine production, current methods for studying IAV kinetics have been limited by the ability to detect all three different RNA species in a scalable manner. Here, we report the development of a novel pipeline using total stranded RNA sequencing (RNA-Seq), which we named influenza virus enumerator of RNA transcripts (InVERT), that allows for the simultaneous quantification of all three RNA species produced by IAV. Using InVERT, we provide a full landscape of the IAV replication kinetics and found that different groups of viral genes follow different kinetics. The segments coding for RNA-dependent RNA polymerase (RdRP) produced more vRNA than mRNA, while some other segments (NP, NS, and hemagglutinin [HA]) consistently made more mRNA than vRNA. vRNA expression levels did not correlate with cRNA expression, suggesting complex regulation of vRNA synthesis. Furthermore, by studying the kinetics of a virus lacking the capacity to generate new polymerase complexes, we found evidence that further supports a model in which cRNA synthesis requires newly synthesized RdRP and that incoming RdRP can only generate mRNA. Overall, InVERT is a powerful tool for quantifying IAV RNA species to elucidate key features of IAV replication.

**IMPORTANCE** Influenza A virus (IAV) is a respiratory pathogen that has caused significant mortality throughout history and remains a global threat to human health. Although much is known about IAV replication, the regulation of IAV replication dynamics is not completely understood. This is due in part to both technical limitations and the complicated replication of the virus, which has a segmented genome and produces three distinct RNA species for each gene segment. We developed a new approach that allows the methodical study of IAV replication kinetics, shedding light on many interesting features of IAV replication biology. This study advances our understanding of the kinetics of IAV replication and will help to facilitate future research in the field.

## Abstract (precis)

Influenza A virus (IAV) is a respiratory pathogen that has caused significant mortality throughout history and remains a global threat to human health. Although much is known about IAV replication, the regulation of IAV replication dynamics is not completely understood.

## INTRODUCTION

Influenza A virus (IAV) is a negative-sense RNA virus with eight gene segments. Each of the viral RNAs (vRNAs) are coated in nucleocapsid protein (NP) and bound by the tripartite viral RNA-dependent-RNA polymerase (RdRp), resulting in eight viral ribonucleoprotein (vRNP) complexes. After entry and uncoating of the virion, vRNP complexes traffic to the nucleus. The RdRp first transcribes vRNA into mRNA, termed primary transcription. Then, the RdRp generates new vRNA through a cRNA intermediate, which is also in complex with an RNP (cRNP) (1, 2). Recent evidence suggests that *de novo* polymerase complexes are required for the virus to switch from transcription to replication (3–5). We have used a single-cycle virus lacking the capacity to generate new PB1 protein to demonstrate that incoming polymerase alone cannot drive any vRNA production (4), further suggesting that replication requires new polymerase complexes functioning in *trans*.

The ability to characterize the kinetics of IAV replication and transcription will help advance our understanding of the biology of IAV replication, which can impact the development of drugs that target viral transcription and replication (6, 7). Many studies have described different kinetics for vRNA, cRNA, and mRNA (8–12). Primer extension was the first assay used to detect all three species of viral RNA and showed a gradation of mRNA, vRNA, and cRNA (9). While this assay has been used to define the fundamental biology of IAV replication, it has several drawbacks, including the need to use radiolabeled probes and limited sensitivity for quantifying levels of each RNA species for all eight segments. More recently, quantitative reverse transcription-PCR (qRT-PCR) assays have been used to assess levels of IAV mRNA, vRNA and cRNA (6, 10, 11). However, high background and low specificity constrain strand- and segment-specific qRT-PCR analyses (13). While there have been improvements to qRT-PCR, such as improved specificity by including a hot start with trehalose to better distinguish between mRNA and cRNA and tagged primers for increased segment specificity (12), evaluating all segments and RNA species from IAV, particularly to assess changes over time, is incredibly laborious. To evaluate each segment kinetically would require at least 24 qRT-PCRs per time point (1 reaction each for mRNA, cRNA, and vRNA times 8 segments, not including reactions for standards). Additionally, differences in primer specificity between segments and between RNA species limit the ability to compare the kinetics of IAV genes, even within a single infection.

To overcome these technical hurdles to rigorously evaluate the viral RNA species kinetics during influenza virus replication, we developed a new method using RNA sequencing and computational calculations we termed influenza virus enumerator of RNA transcripts (InVERT). This pipeline takes advantage of the differences in the 3′ end of the positive-sense viral RNA species. Our strategy allows for quantification of all IAV genes and RNA species independent of primer bias. We validate our findings by comparing the level of mRNA through our new approach against traditional poly(A)-based sequencing and analysis. By distinguishing the kinetics of all three viral species, we found that different RNA species follow different kinetic profiles, as follows: vRNA levels increase during the entire course of infection, cRNA levels increase and then stabilize, and mRNAs reach a peak and then decline. We also quantified the kinetics of each virus segment and discovered segment-specific heterogeneity in replication kinetics. Specifically, the RdRP genes (PB1, PB2, and PA) produce significantly lower levels of mRNA than vRNA, whereas several other genes, such as NP, HA, and NS, consistently generate a higher level of mRNA than vRNA. Interestingly, the change in the level of vRNAs is not necessarily correlated with its temporary template cRNAs. By employing InVERT to study the kinetics of an engineered IAV lacking PB1, we also found that without newly synthesized PB1 proteins, only mRNAs were detected. This finding indicates that incoming RdRPs cannot trigger cRNA synthesis to initiate viral genome replication. Overall, using this novel InVERT pipeline, we have gained insight into how IAV regulates its replication kinetics between different viral segments and RNA species.

## RESULTS

### A method to distinguish influenza virus vRNA, cRNA, and mRNA.

**FIG 1:**
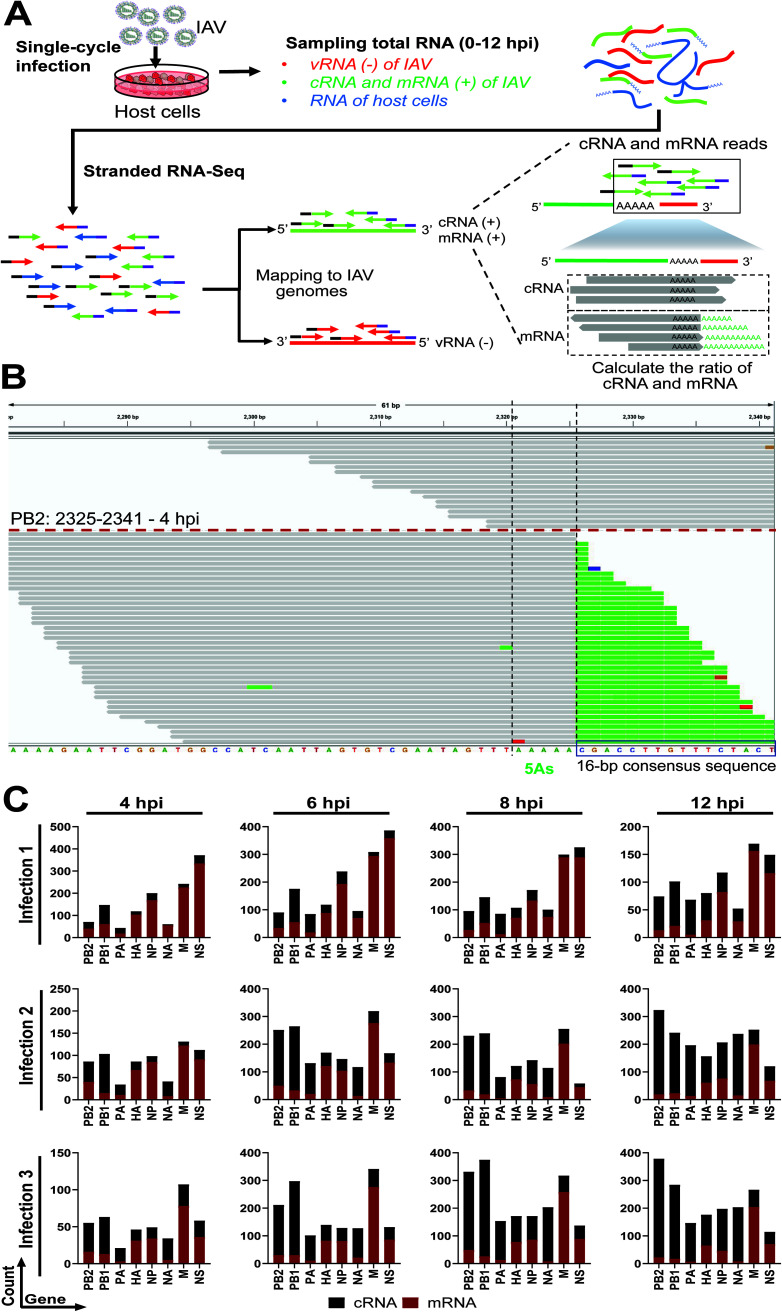
Influenza virus enumerator of RNA transcripts (InVERT) to study IAV replication kinetics. (A) Schematics of the experimental pipeline. MDCK cells were infected with IAV PR8 at a multiplicity of infection (MOI) of 0.5. Total RNA (which includes viral negative-sense RNA [red], viral positive-sense RNA [green], and host cell RNA [blue]) was harvested at different time points after infection. Total RNA was then subjected to stranded-RNA sequencing. Paired-end reads were mapped to MDCK and IAV genomes for quantification. The ratio of cRNA to mRNA was determined by dividing the number of reads that mapped to the 16-nucleotide (nt) consensus sequencing for cRNAs by the number of reads that have the poly(A) tail after the 5 As (mRNA). The expression levels of cRNA and mRNA were calculated from the cRNA:mRNA ratio and the total expression value of positive-sense RNAs. (B) Integrative Genomics Viewer (IGV) visualization of reads that mapped either to cRNA or mRNA of PB2 at 4 h postinfection (hpi). The 5 As was highlighted between the 2 black dotted lines (corresponding to the 2,321 to 2,325 bp coordinates), followed by the 16-nt consensus sequence (framed in the black box). Reads mapped to cRNA are represented as gray bars above the red dotted line, and reads mapped to mRNA are presented as gray bars with the poly(A) sequence below the red dotted line. Different colors on the gray bars represent mismatched nucleotides to the reference (green, A; red, T; orange, G; blue, C). (C) Number of RNA-Seq read counts mapped to cRNAs versus mRNAs at the 3′ end. Number of RNA-Seq reads at the 3′ end that are differentiated into cRNA (black) or mRNA (red) reads from 3 independent infections of PR8 into MDCK cells at different time points were plotted.

Currently, methods for evaluating IAV replication can be time consuming and laborious. Given the expanded use, reduced cost, and ease of deep sequencing technology, we developed a pipeline that allows for scalable analysis of IAV replication kinetics (Fig. 1A). Total RNA from IAV infected cells at different time points was analyzed by total stranded RNA sequencing (RNA-Seq) so that directionality of RNA reads was maintained. Reads were then mapped to viral and host genomes, during which reads from the negative-sense (vRNA) and positive-sense (cRNA and mRNA) RNAs could be identified. To discriminate between cRNAs and mRNAs, we relied on the differences between these RNAs at the 3′ end. Each viral genome segment incorporates a conserved sequence of 5 or 6 adenosines (referred to here as 5 As). During transcription, this sequence is used by the RdRP for adding the 3′ poly(A) tails via a “stuttering” mechanism (14, 15). For cRNA, following these 5 As are 16 nucleotides (nt) of cRNA-specific sequence, in which 13 bp are conserved across all viral segments (Table 2). Reads that contain poly(A) tails after the 5 As indicate mRNAs, while reads with conserved consensus sequence after the 5 As denote cRNAs (Fig. 1B). We established a pipeline we termed InVERT to extract all of the reads that extend over the 5 As, counted the numbers of reads that mapped either to mRNA or cRNA, and then used their ratio to calculate the level of each RNA species from the total expression value. Figure 1B is a representation of reads that were extracted for the analysis. Overall, there is a clear and consistent separation between cRNA and mRNA reads (Fig. 1B and C).

**TABLE 2:**
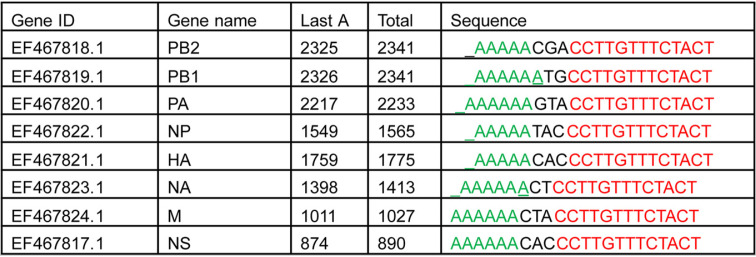
The consensus sequences and locations of poly(A) for 8 viral segments of IAV PR8^*a*^

a Table lists sequence of the 5 As (“Sequence”) and the consensus sequence for each gene identifier (GenBank accession number)/gene name, as well as the genome coordinates of the last A in the 5 As (“Last A”) out of the total length (“Total”). Green, 5As; red, consensus sequence.

### Comparison of the mRNA kinetics determined from InVERT against poly(A)-based mRNA sequencing.

**FIG 2:**
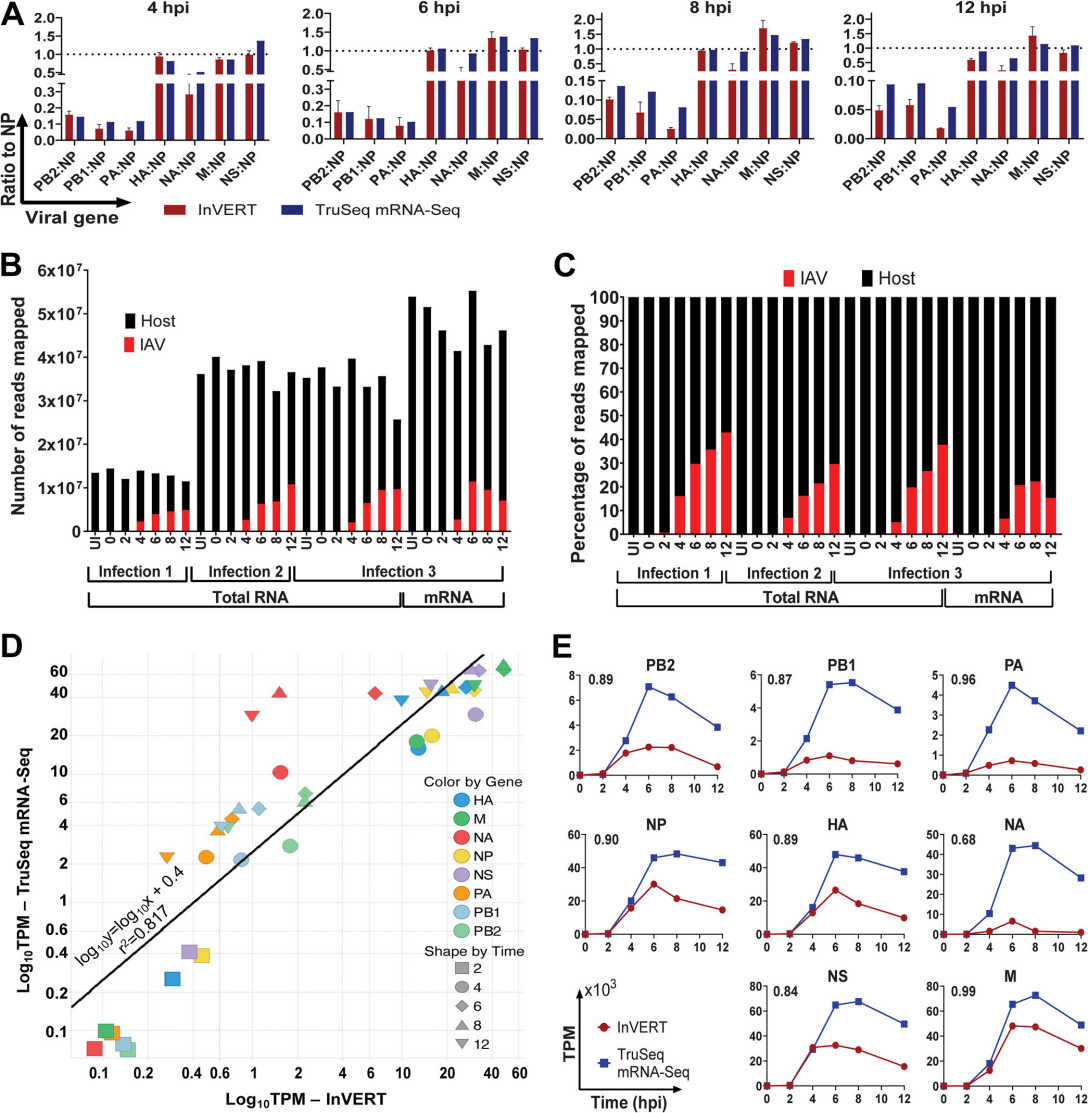
Comparison between mRNA expression levels identified by InVERT and by TruSeq mRNA-Seq. (A) Relative viral mRNA expression levels (normalized to NP) determined by either InVERT (red) or TruSeq mRNA-Seq (blue) at different time points postinfection. RNA from the same infection was used as input for both TruSeq mRNA-Seq and InVERT methods. (B) Number of reads mapped to IAV (red) or to the host genome (black) for all RNA-Seq samples from IAV PR8 infection. Each column represents the number of reads mapped from one RNA-Seq sample, either from total RNA-Seq (for all three infections) or from mRNA-Seq (for only the third infection). Each sequencing sample is from one time point (uninfected [UI], 0, 2, 4, 6, 8, and 12 hpi) out of three infections. (C) Percentage of reads mapped to either IAV (red) or host (black) genomes from all RNA-Seq samples from IAV PR8 infection. More information for these sequencing samples can be found in Table 1. (D) Correlation between mRNA expression determined by InVERT and TruSeq mRNA Seq. The black line indicates linear regression by least-squares approximation. (E) mRNA kinetics determined either by InVERT (round red) or by TruSeq mRNA-Seq (square blue). Data are plotted by transcripts per kilobase per million (TPM) (10^3^) against time postinfection. The number on the top left corner of each graph indicates the Pearson correlation coefficient between the two detection methods.

To validate our pipeline, we compared the level of relative viral mRNAs quantified by InVERT to mRNA independently quantified by poly(A)-based RNA sequencing (mRNA-Seq). Importantly, the RNA used for each of these methods was generated from one experiment, eliminating any differences due to viral stocks, kits, or buffers, or to timing of infections or harvests, allowing us to directly compare the sequencing methods. At each time point, the transcripts per kilobase per million (TPM) of all viral mRNAs were normalized to that of NP for both InVERT and mRNA-Seq. The relative expression levels of all viral mRNAs, except for NA and PA, obtained by total stranded RNA-Seq (SMARTer-Seq) and InVERT were comparable with those measured by direct mRNA sequencing (Fig. 2A). The relative NA and PA mRNA levels determined by InVERT were lower than those determined by mRNA-Seq at 4, 8, and 12 hours postinfection (hpi). In general, the RdRP mRNAs were more than 5-fold lower than NP mRNA by both methods of determination, while all of the other viral mRNAs were present in the same range as NP in all samples. The consistency of the relative expression value of viral mRNAs between the two methods demonstrated that using InVERT with total stranded RNA-Seq can serve as a reliable tool to study IAV replication kinetics.

The direct comparison of the raw expression values from the two sequencing methods was hindered by the many technical differences that impact absolute read numbers, including library preparation, rRNA reduction, sequencing flow cells, the number of reads mapped, etc. The two methods further differ in that the total stranded SMARTer-Seq covers all of the host and viral RNA species (vRNA, cRNA, and mRNA), while TruSeq mRNA sequencing assays only mRNA (Table 1 and Fig. 2B and C). Hence, mRNA TPM values determined by TruSeq mRNA-Seq are higher than those determined from SMARTer-Seq (Fig. 2E). At early time points, when transcription dominates, the mRNA TPMs between the two methods are well correlated. However, there is some divergence at later time points, when vRNA levels increased exponentially. The increase in vRNA alters the RNA proportions in total RNA pools for total RNA-Seq and is one contributor to the discrepancy between the two sequencing methods (Fig. 2B and C). Nonetheless, the TPM determined by the two methods had a high correlation (*R*-squared = 0.817) (Fig. 2D). Overall, the two methods reveal almost identical kinetics for all genome segments (Fig. 2E). Most segments showed a rapid increase in viral mRNA followed by a moderate decrease. Pearson correlation showed a high degree of similarity of mRNA kinetics determined between the two methods (Pearson correlation coefficients of >0.8 for all of the genes except NA). Together, these results demonstrate that InVERT can faithfully identify IAV transcription kinetics.

**TABLE 1:** Library preparation and RNA-Seq technology used for analysis of IAV PR8 infections of MDCK cells

Parameter	Infection 1, Seq R1	Infection 2, Seq R2	Infection 3	
			Seq R3	Seq R4
Library prep method	Clontech Pico SMARTer stranded total RNA-Seq kit v2	Clontech Pico SMARTer stranded total RNA-Seq kit v2	Clontech Pico SMARTer stranded total RNA-Seq kit v2	Illumina TruSeq mRNA
Sequencing method	HiSeq 2500 high output, 50-bp PE^*a*^ (v4 chemistry)	NovaSeq SPrime 2 × 50-bp PE	NovaSeq SPrime 2 × 50-bp PE	NovaSeq S1 2 × 150-bp PE
Approximate depth (million reads/sample)	16.9	26.8	26.8	20.3

a PE, paired end.

### Using InVERT to determine a kinetic profile of IAV replication.

**FIG 3:**
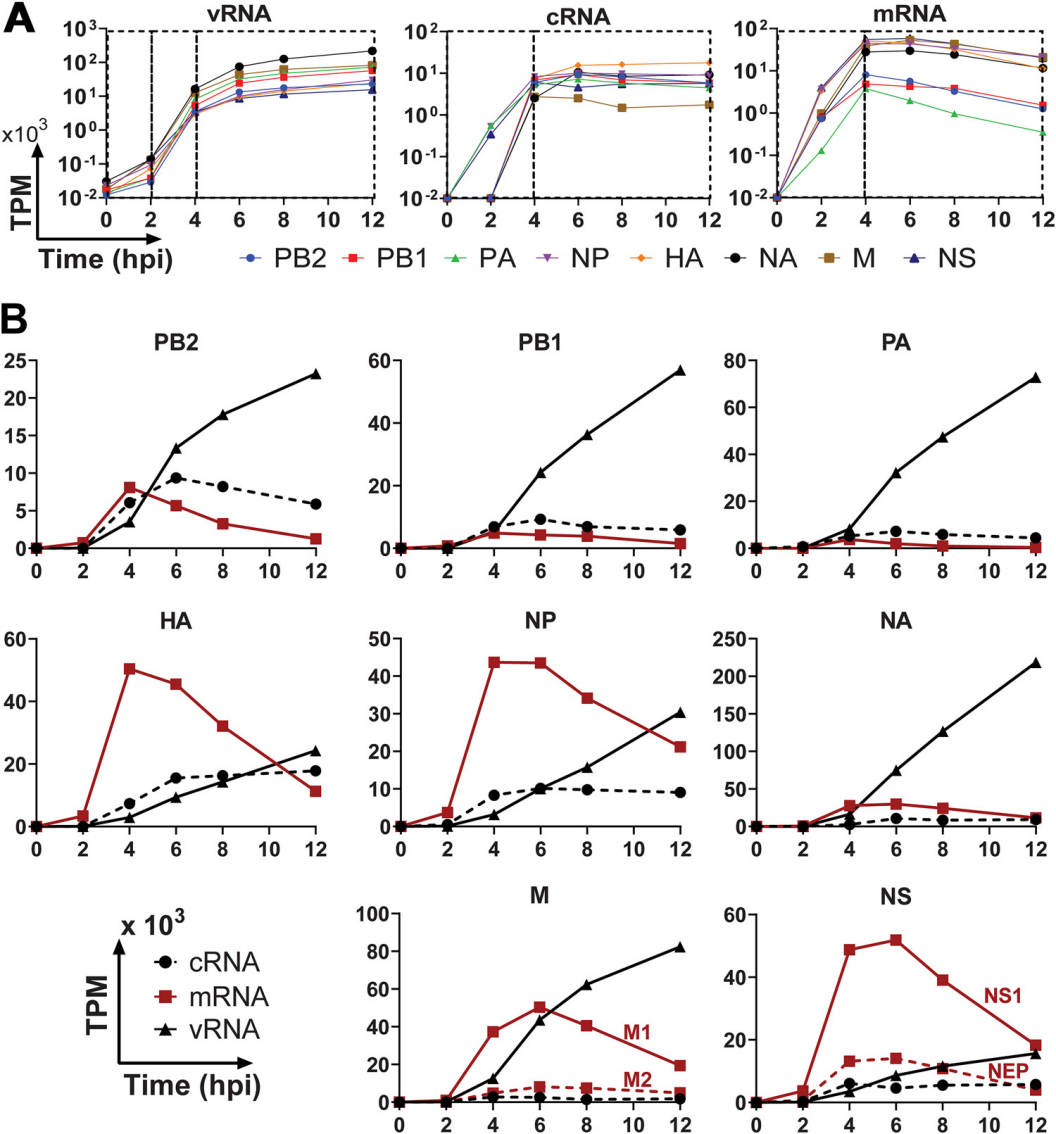
Kinetic profile of IAV RNA species during infection. (A) Different phases of the IAV single-cycle replication. Kinetic profiles of IAV infection were plotted by RNA species. Each graph plotted TPM value (in log scale) against infection time for all viral genes (left, vRNA; middle, cRNA; right, mRNA). Each black box highlights one phase of replication, in which the change in RNA expression level is consistent. (B) Kinetic profiles of IAV infection by gene segments. Each panel in this trellis graph shows the kinetics of vRNA (▴), cRNA (●), and mRNA (▪) of one viral segment as a function of time postinfection. Data for two additional experimental replicate infections are shown in Fig. S2 in the supplemental material.

We used InVERT to evaluate the replication kinetics of all three IAV RNA species for all IAV segments (Fig. 3A). From 0 to 2 hpi, rapid increase of mRNA with no increase in vRNA indicated an active phase of primary transcription being driven by incoming RdRPs. From 2 to 4 hpi, active replication was reflected in the marked increase in vRNA and cRNA production along with stabilizing levels of mRNA. Starting at 4 hpi, vRNA continued to increase, cRNA levels stabilized, and mRNA levels transitioned to a gradual decrease. This could be due to degradation and/or a lower rate of transcription. These data support previously reported IAV kinetics, both in mRNA (12, 16) and in protein (17).

**FIG 4:**
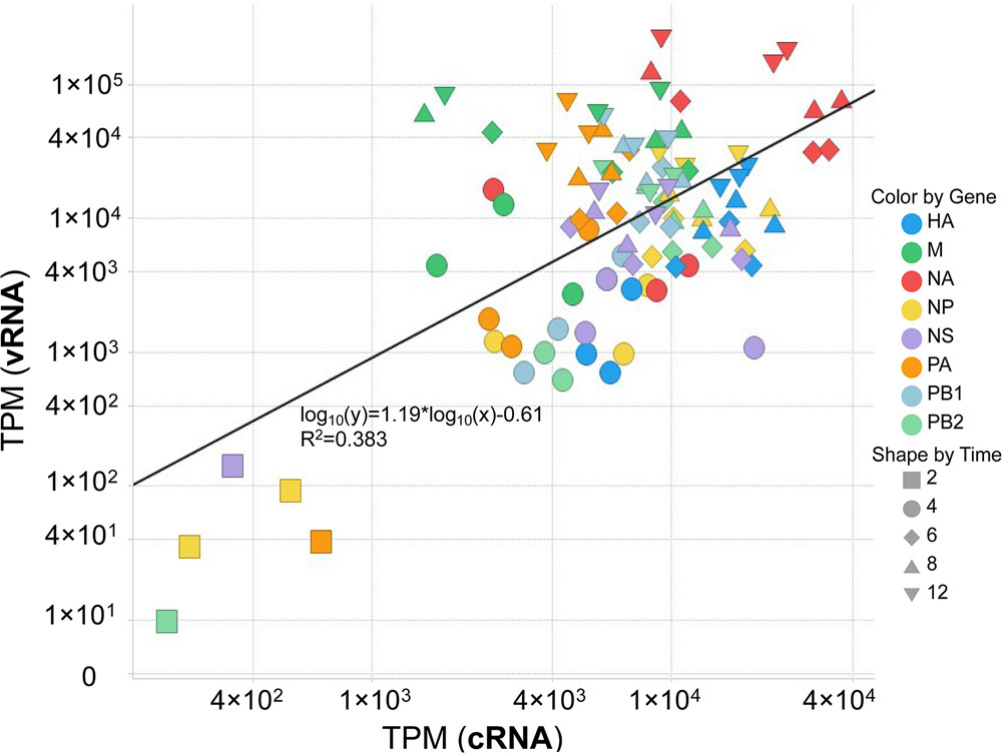
Viral cRNA dynamics do not have a linear correlation to vRNA dynamics. Scatterplot of cRNA and vRNA expression. Each data point represents expression of vRNA versus cRNA at one time point (▪, 2 hpi; ●, 4 hpi; ♦, 6 hpi; ▴, 8 hpi; ▾, 12 hpi) for one gene (color coded). The regression line (black continuous line) is acquired from least-squares regression approximation (*R*^2^ = 0.383). Data are combined from three independent infections.

We also noted kinetic patterns among RNA species of different segments (Fig. 3B; see also Fig. S2 in the supplemental material). The mRNA from the polymerase components PB2, PB1, and PA are observed at significantly lower levels than their corresponding vRNAs. In contrast, HA, NP, and NS had higher levels of mRNA compared to vRNAs, which reflected the abundance of these viral proteins in infected cells (18). The abundance levels of vRNA of different segments differ by more than 10-fold with the highest being NA and the lowest NS across all three independent infections. Levels of PB2 vRNA were 2 to 3 times lower than that of either PB1 and PA at 12 hpi. The level of cRNAs did not strongly correlate to the level of vRNAs, even though cRNAs are the template of vRNAs (Fig. 4). At later time points postinfection, cRNA levels remained stable, while levels of vRNAs continued to increase exponentially.

**FIG 5:**
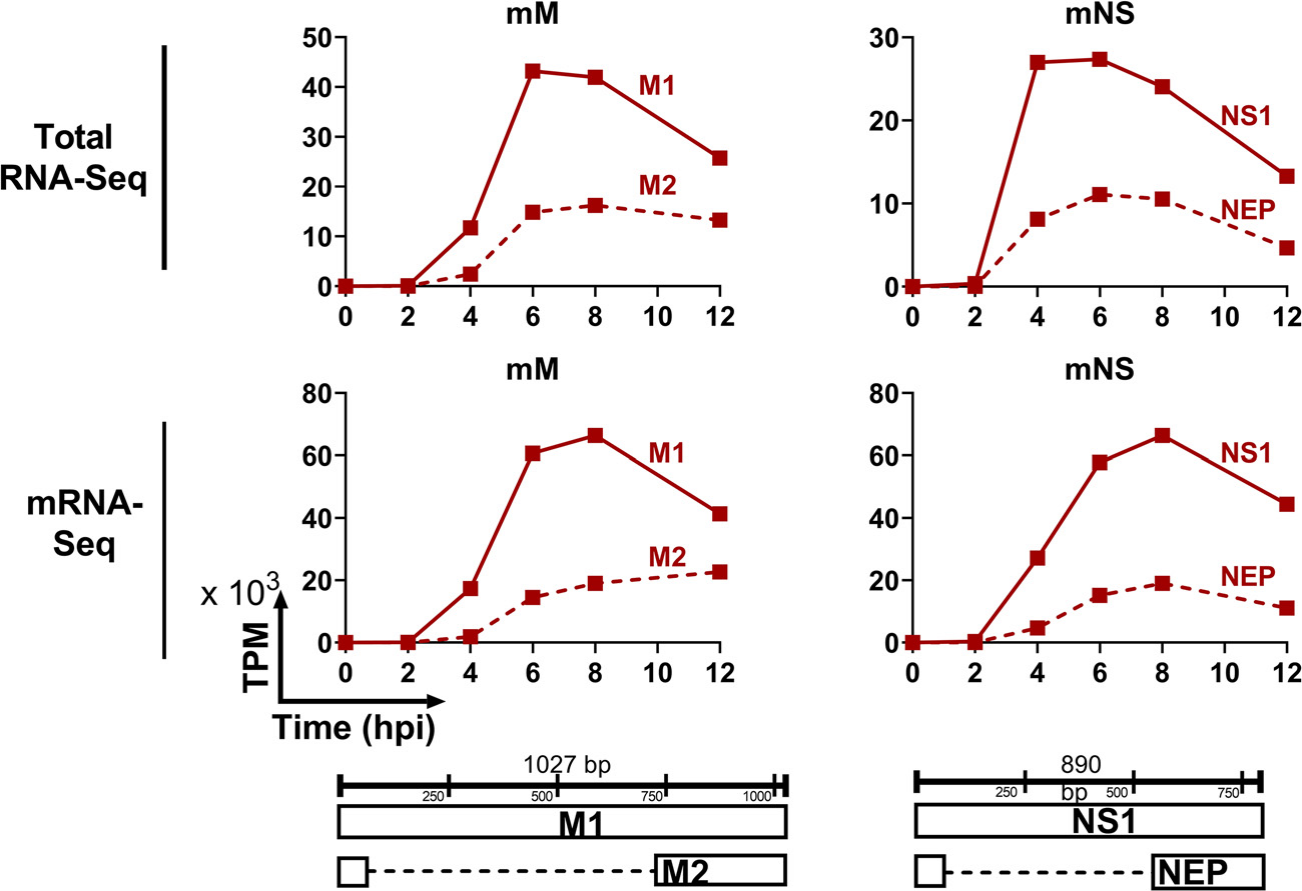
Kinetic profile of mRNAs of alternative spliced genes. Each panel in this trellis graph shows the kinetics of M (left) or NS (right) mRNA determined from total RNA-Seq (upper panels) or mRNA-Seq (lower panels). Transcript expression of spliced isoform (M2 and NEP) (solid lines) and unspliced isoform (dotted lines) are plotted as TPM (×10^3^) again time postinfection.

The segments NS and M each generate two mRNAs from a single vRNA template via splicing, namely NS1 and NEP from NS and M1 and M2 from M. Positive-sense RNAs from total RNA-Seq contain both cRNAs and mRNAs, so traditional alternative splicing isoform quantification tools would also count cRNA reads as unspliced transcripts. We further adapted the pipeline to be able to distinguish between the unspliced and spliced variants of M and NS. We developed an algorithm to quantify splice isoform transcripts based on the ratio of spliced to unspliced reads at the splice junctions. We then used the ratio of cRNA to mRNA to determine the total read counts of spliced and unspliced mRNA. Consistent with previous reports (19–21), the unspliced transcripts (M1 and NS1) had higher expression levels compared to spliced transcript (M2 and NEP) (Fig. 3 and 5 and Fig. S2). In contrast to the decrease in M1 at late stage of infection, M2 expression level remained steady. To validate our pipeline, we also calculated the kinetics of NS1, NEP, M1, and M2 using traditional poly(A)-based mRNA sequencing. These data demonstrated similar kinetic patterns of NS1/NEP and M1/M2 compared to our new approach (Fig. 5). Together, these data demonstrate that InVERT can also be applied to identify alternatively spliced products of IAV.

### The kinetic profile of the delta-PB1 into A549 reveals the mechanism of cRNA synthesis.

**FIG 6:**
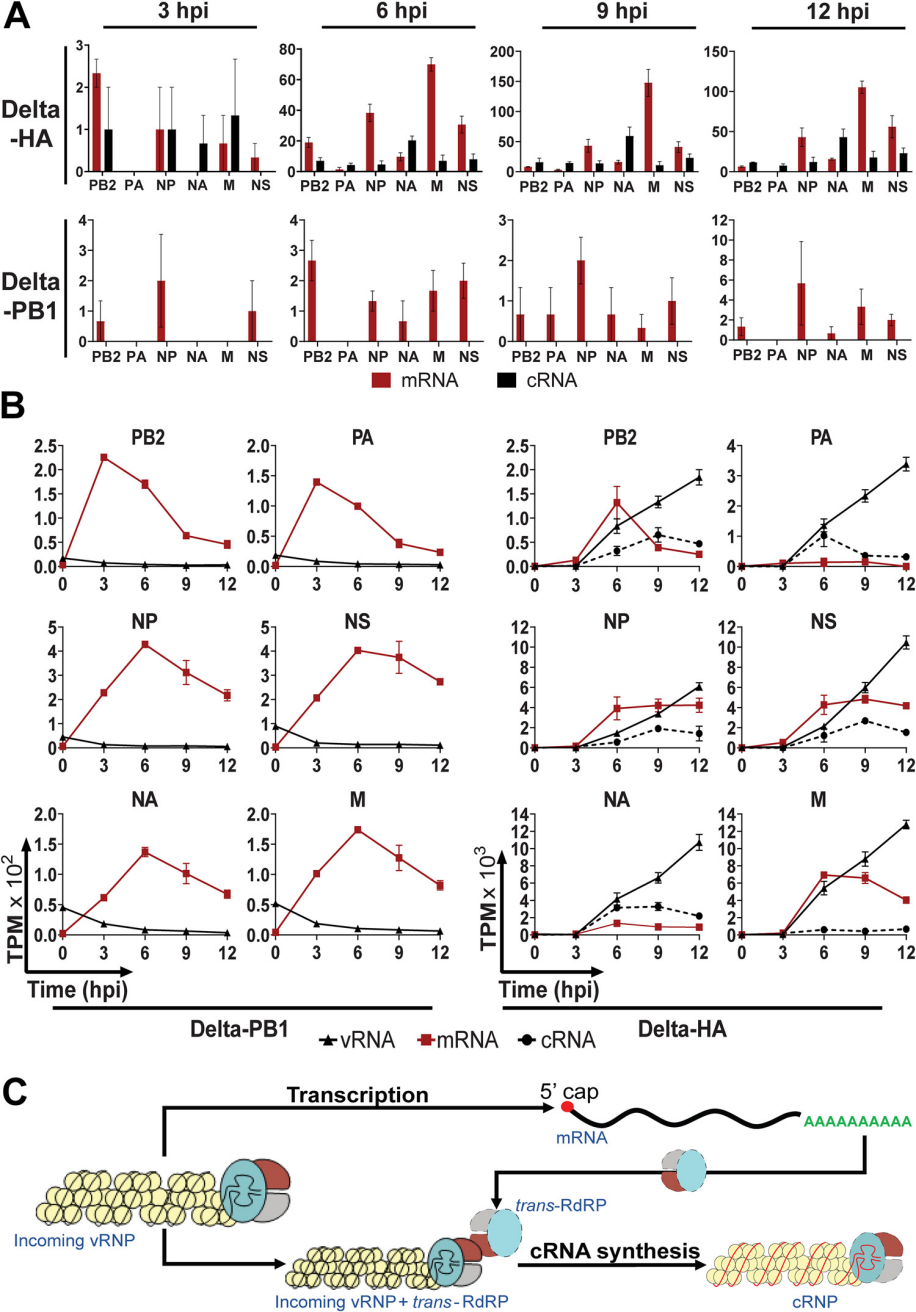
Kinetics of delta-PB1 virus demonstrates that incoming polymerase cannot initiate cRNA synthesis. (A) Number of RNA-Seq read counts mapped to cRNAs versus mRNAs at the 3′ end. Number of RNA-Seq reads at the 3′ end that are differentiated into cRNA (black) or mRNA (red) reads from delta-HA virus (top row) and delta-PB1 virus (bottom row). Error bar indicates standard error of the mean (SEM). (B) Comparison replication kinetics of delta-HA and delta-PB1 viruses. Each panel in this trellis graph shows the kinetics of vRNA (▴), cRNA (●), mRNA (▪) of one viral segment along with time postinfection. Each graph plotted the average value of the three infections. Error bar indicates SEM. (C) Model for cRNA synthesis initiation. Incoming viral ribonucleoproteins (vRNPs) can initiate primary transcription to make mRNA for new RdRP protein. These newly synthesize RdRPs (*trans*-RdRPs) play roles in activating cRNA synthesis, potentially through the same *trans*-activating mechanism as in vRNA synthesis (3).

Upon the entry of IAV into cells, the RdRPs packaged within the virion use the vRNA template to drive both primary transcription of mRNA and replication to generate new vRNA. It has been suggested that vRNA synthesis requires an additional RdRP to participate in *trans* (3). However, how transcription switches to replication and whether incoming RdRPs alone can generate cRNAs has not been determined. With the ability to sensitively differentiate cRNA from mRNA, we addressed this question by examining kinetics of a virus lacking PB1 vRNA (delta-PB1) which cannot generate new PB1 (4). As a control, we used a virus lacking HA vRNA (delta-HA), which is capable of RdRP-mediated transcription and replication. A549 cells were infected with either delta-HA or delta-PB1. In the absence of the newly synthesized RdRP, delta-PB1 was able to drive mRNA transcription, albeit at lower levels than transcription driven by delta-HA (Fig. 6A and B). The vRNAs detected at 0 hpi, which are likely from incoming vRNPs, dropped to nearly undetectable levels by 3 hpi, indicating the absence of vRNA synthesis. Delta-HA, which is capable of synthesizing new RdRPs, drove high mRNA expression of all virus genes, and importantly, cRNAs and vRNAs were also synthesized and followed similar kinetics to those of the wild-type influenza A/Puerto Rico/8/34 (PR8) virus. Conversely, no cRNA was detected in delta-PB1-infected cells (Fig. 6A and B). These data suggest that incoming virus polymerase cannot generate cRNA and support the model proposing that IAV genome replication relies on *trans*-activating RdRP (2, 3) and *de novo* RdRPs are necessary to initiate cRNA synthesis.

## DISCUSSION

Here, we report a pipeline to quantify the three species of IAV RNAs (vRNA, cRNA, and mRNA) from all genome segments using stranded total RNA-Seq. Using this pipeline, we demonstrate distinct dynamics of vRNA, cRNA, and mRNA of different viral genes upon IAV infection. The disparate detection of IAV RNA species demonstrates highly dynamic transcription and replication kinetics that can vary across segments. By applying the pipeline to study the replication kinetics of delta-PB1 virus, we validated that without *de novo* PB1 protein, cRNA synthesis was not initiated. Compared to viral RNA quantification based on qRT-PCR using specific primers, our pipeline uses RNA-Seq, which does not rely on viral gene-specific primers for either reverse transcription or PCR and thus minimizes primer biases. Additionally, quantification of IAV RNAs by InVERT is more direct and straightforward and does not need to be referenced to housekeeping genes or *in vitro*-applied RNA standards (see Fig. S3 in the supplemental material). As RNA-Seq has become low cost and readily accessible, InVERT can serve as a strong alternative method for studying IAV replication kinetics.

As InVERT relies on the sequence difference of reads mapped to the 3′ end of RNA to distinguish cRNAs and mRNAs, a sufficient coverage of the 3′ end of a transcript is critical for confident calculation of the cRNA/mRNA ratio. The number of reads mapped to the influenza genome changes according to the time point, the multiplicity of infection (MOI), and the quality of the RNA-Seq run. At earlier time points during infection (before 4 hpi), the low number of viral reads leads and lower sequencing coverage and could affect accuracy (see Fig. S1 and S8 in the supplemental material). This can be improved by increasing sequencing depth. For samples starting from 4 hpi, the coverage of reads is high enough to distinguish between cRNA and mRNA (Fig. 1B and C and Fig. 6A). Based on our established protocol and results, we recommend having at least 1 million reads mapped to influenza virus to ensure the accuracy of the quantification. The number of reads mapped to influenza changes according to the MOI of infection, the time postinfection, and the quality of the RNA-Seq run. For instance, assuming that 8% of total reads are flu-derived reads, and 65% of the total reads sequenced mapped into genomes, the sequencing depth should be at least 19.23 million reads per sample to have the coverage of 1 million viral reads. Because this analysis relies on the 3′ end of the positive-sense viral RNA, it is possible that it will capture the complement of mini viral RNAs and count them as cRNA, artificially inflating cRNA numbers (22). mRNAs of IAV also differ from cRNAs by the capping sequence at the 5′ end that the RdRPs snatch from host cell mRNAs. The PA protein in the RdRP cleaves 10 to 13 nucleotides from cellular mRNAs and uses this sequencing to prime the transcription, whereas cRNA synthesis is a primer-independent process (2). Although it might be tempting to use this difference to distinguish cRNA and mRNA in deep-sequencing data sets, a diverse repertoire of the relatively short capping sequences from hosts makes their identification difficult (23).

The RNA profile assessed by our pipeline provides a reliable picture of the replication dynamics of all viral genes except for some uncertainty in mRNA levels of NA and PA because of the discrepancy with direct mRNA sequencing. Lower mRNA expression of NA with similar dynamics has also been reported using qRT-PCR (12), suggesting that the possible underestimation of NA might be due to the properties of this mRNA or its interaction with other RNA species. The relative abundance of the three types of viral RNAs and their dynamics differ among the eight segments. The different kinetics and differing abundances suggest that replication of different segments is regulated differently. Lower levels of RdRP mRNAs compared to those of the other IAV genes is well correlated with the protein dynamics, in which RdRP protein levels are also the lowest across infection (18). Interestingly, cRNA dynamics did not appear to have any linear correlation with the vRNA dynamics (Fig. 4), indicating that the concentration of cRNA is not the sole determinant regulation of vRNA expression levels. This was also seen in previous reports, in which a higher level of cRNA expression did not correspond to an elevated vRNA expression (8, 12). The regulation of vRNA dynamics therefore likely depends on additional elements such as host factors (24), interaction of cRNA with RdRP, and interactions between RNPs (25, 26). While the “perfect model” of infection includes packaging one of each segment into new viruses, the reality is that does not happen all or even most of the time, and many virions fail to package at least one segment (27–29). In our analysis, PB2 had one of the lowest levels of vRNA, and this segment was found to be the segment most frequently missing in single-cell RNA sequencing analyses (29). These data suggest that vRNA abundance, in addition to other factors, could impact the packaging of complete virions.

A previously proposed stabilization model asserts that earlier during influenza infection, cRNAs and mRNAs are both synthesized, but cRNAs are degraded until PB2, PB1, PA, and NP are abundant enough to stabilize cRNA as cRNP (5, 30). NP does not affect the initiation or termination of replication or transcription, but it is critical for cRNA stabilization, and NP-RNA binding and oligomerization of NP are essential for genome replication (31, 32). Replication of vRNAs from cRNA templates was shown to require a *trans*-activating RdRP (3). As vRNA and cRNA synthesis are both primer independent, cRNA synthesis was thus speculated to need a *trans*-RdRP to be initiated. Unlike earlier studies that mostly used indirect assays on purified RNPs, our delta-PB1 virus infection experiment provides more direct evidence to support that model. The time course data of delta-PB1 virus infection show that no cRNA was generated without new RdRP protein synthesis. cRNA has previously been detected at 2 hpi but at a very low level compared to that of mRNA (12), similar to what we observed here using InVERT on PR8-infected cells. We speculate that the transition from transcription to replication of influenza virus is determined by the levels of newly synthesized RdRP made to initiate the cRNA synthesis. However, *trans*-RdRP level might not be the sole factor governing the transition between transcription and replication. *Trans*-RdRP protein level and cRNA stabilization by NP binding could both contribute to the transitioning effects. How *trans*-RdRP interacts with the incoming vRNAs to synthesize new cRNAs remains unclear.

Taken together, this study reports a new method to quantitatively study the kinetics of influenza-derived RNA. The method is robust and provides a more complete picture of influenza vRNA, cRNA, and mRNA kinetics. This revealed groups of influenza genes with distinct patterns of expression, suggesting segment-specific regulation during replication. Armed by the pipeline, we were able to provide additional evidence supporting the hypothesis that *trans*-RdRP is required to initiate cRNA synthesis.

## MATERIALS AND METHODS

### Cells and virus.

Madin-Darby canine kidney (MDCK) and human lung adenocarcinoma epithelial A549 cells were maintained in Dulbecco’s modified Eagle medium (DMEM) supplemented with 10% fetal bovine serum (FBS). Recombinant influenza A/Puerto Rico/8/34 (PR8) was rescued by plasmid-based transfection, plaque purified, and propagated in embryonic chicken eggs. The single-cycle viruses (delta-HA and delta-PB1) in the PR8 backbone were created by replacing the coding sequence of HA and PB1 by mCherry and amplified in MDCK cells engineered to express HA and PB1, respectively, as previously described (4). Viral titers were determined by plaque assay in MDCK cells.

### Influenza virus infections.

MDCK cells were infected with IAV PR8 (MOI = 0.5) and incubated at 4°C for 30 min to allow the virus to adsorb to the cell surface but not to enter the cytosol. Synchronized IAV infection was initiated by shifting the temperature to 37°C. Cells were harvested at 0, 2, 4, 6, 8, and 12 h post temperature shift for RNA-Seq to quantify the cellular RNA and viral RNA levels. Uninfected MDCK cells were also collected for a negative control. Three independent infections at each time point were performed. Infections of single-cycle viruses (delta-HA and delta-PB1) in A549 cells were as previously described (4).

### RNA library preparation and sequencing.

RNA from three independent time series infections of IAV PR8 in MDCK cells were extracted using the RNeasy minikit (Qiagen). RNA quality was assessed by Agilent TapeStation or Agilent Bioanalyzer. All RNA samples had RNA integrity score of 9.0 or higher. To assay both positive- and negative-sense RNAs, total RNA was prepped using the Clontech Pico SMARTer stranded total RNA-Seq kit v2, in which strand orientation of the original RNA was preserved by the template-switching reactions. The cDNA library, with an insert size of ∼200 bp, was then subjected to sequencing, either on the HiSeq 2500 High Output using v4 chemistry (infection 1) or on the NovaSeq SPrime sequencing platform (infections 2 and 3) (Table 1). For the third infection, a portion of the RNA was also prepped by Illumina TruSeq stranded mRNA prep kit, which will isolate mRNA only by oligo(dT) beads and sequenced on the NovaSeq S1 platform. At least 20 million 150-bp paired-end (PE) reads were generated per sample. Details on library preparation and sequencing can be found in Table 1. For single-cycle virus infections (delta-HA and delta-PB1) of A549 cells, RNA was extracted using TRIzol, prepared for sequencing using the stranded total RNA v2 Pico masmmalian kit, and sequenced using NovaSeq (Illumina) as described in Fay et al. (4). The raw data, reported as fastq files, were deposited at NCBI-GEO under series GSE162281.

### Data analysis pipeline for quantifying gene expression level.

**(i) Reference genome and annotation files.** Reference genomes, namely CanFam3.1 for MDCK cells or GRCh38.p13 for A549 cells, were downloaded from Ensembl. The IAV PR8 genome was assembled by combining complete sequences for all eight genome segments from the Influenza Research Database (https://www.fludb.org) with NCBI taxon identifier (ID) 211044. The IAV delta-HA and delta-PB1 virus genomes were assembled by concatenating 6 genome segments of the IAV PR8 (excluding HA and PB1). The host genome and IAV genome were concatenated into one combined reference genome that was used for mapping. The annotation file for IAV PR8, delta-HA, or delta-PB1 was curated such that each genome segment was annotated for negative-sense and positive-sense RNA. The stranded library prep preserved the orientation of all reads.

### (ii) Preprocessing and mapping.

All of the raw RNA-Seq reads were processed with adapter trimming and low-quality base removal by Trimmomatic (33) and checked for quality using FASTQC (34). All trimmed reads with a minimum read length of 38 bp and average quality per base greater than 30 were mapped into the combined genome of host plus IAV by STAR v2.5.3a (35) (see Box 1 in the Supplemental Methods). Separation of reads that were mapped into sense or antisense strands was performed using SAMtools (see Box 2 in the Supplemental Methods) (36).

### Calculating expression level of each RNA species.

The expression levels of all of the genes, including both host and viral genes, was quantified first by Cufflinks (37) to count the number of reads that are assigned to annotated features (e.g., gene or transcript) in the annotation file and calculate fragments per kilobase of transcript per million mapped reads (FPKM) value for each feature. FPKM of viral negative-sense RNA (vRNA) or positive-sense RNA (cRNA and mRNA) was determined by providing an annotation file that defines RNA strandedness during the quantification. The FPKM values from Cufflinks were converted into transcripts per kilobase per million (TPM).

Viral cRNAs and mRNAs were distinguished using SAMtools (36). cRNA and mRNA are distinguished based on the sequence following five adenosines (5 As) at the 3′ end of the gene. During transcription of mRNA, the 5A sequence is extended to form the poly(A) tail, whereas all IAV cRNAs contain a 16-nucleotide (nt) sequence—13 of which are conserved across segments—following the 5 As. For every individual segment in each sample, the pipeline counts the number of reads that contain the 5 As and at least two following nucleotides completely matching the 16-nt consensus sequence as cRNA reads. Reads that contain more than two As extended after 5 As are called as mRNA reads. The coverage of these reads, which is the total number of reads that contain poly(A) tails for mRNA and reads that overlap the 16-nt consensus sequence for cRNA, was calculated using SAMtools. The ratio of mRNA to cRNA was calculated (see Box 3 in the Supplemental Methods). The number of reads that mapped into either cRNAs or mRNAs can be visualized using Integrative Genomics Viewer (IGV) (38).

For alternatively spliced genes (M and NS), the expression levels of spliced (M2 and NEP) and unspliced (M1 and NS1) isoforms were calculated using the ratio of the spliced to unspliced isoforms and total TPM of mRNA. The ratio of the spliced to unspliced isoforms was determined from the difference in coverage at the splice junctions, including both 5′ and 3′ splice sites. Unspliced mRNA reads were calculated as the total mRNA reads minus the spliced reads. Total mRNA reads at the splice sites were calculated from the total positive-sense RNA-Seq reads using the ratio of mRNA to cRNA determined by InVERT. The spliced to unspliced ratio was then used to calculate the adjusted TPM of total mRNA to calibrate for the shorter length of the spliced transcripts. The spliced to unspliced isoform ratio and the adjusted TPM of total mRNA were used to determine the expression level of spliced and unspliced mRNAs (see Box 4 in the Supplemental Methods).

### Statistical analysis.

TPM values of viral mRNAs determined by InVERT and mRNA-Seq were plotted against each other using Spotfire (TIBCO Software, Inc.), and linear regression was established by least-squares approximation. Pearson correlation was calculated using GraphPad Prism 8 to estimate the similarity between mRNA kinetics determined by TruSeq mRNA-Seq versus by those determined by InVERT. Error bars indicate standard error of the mean (SEM).

### Data availability.

Raw data, reported as fastq files, have been deposited at NCBI-GEO under series GSE162281.

## Supplementary Material

Supplemental file 1
