# Supplementary material for: Segment-Specific Kinetics of mRNA, cRNA, and vRNA Accumulation during Influenza Virus Infection
Source: J Virol. 2021 Apr 26;95(10):e02102-20. doi: 10.1128/JVI.02102-20 (PMC8139675; doi:10.1128/JVI.02102-20)
Supplement: Supplemental file 1 [file JVI.02102-20-s0001.pdf]

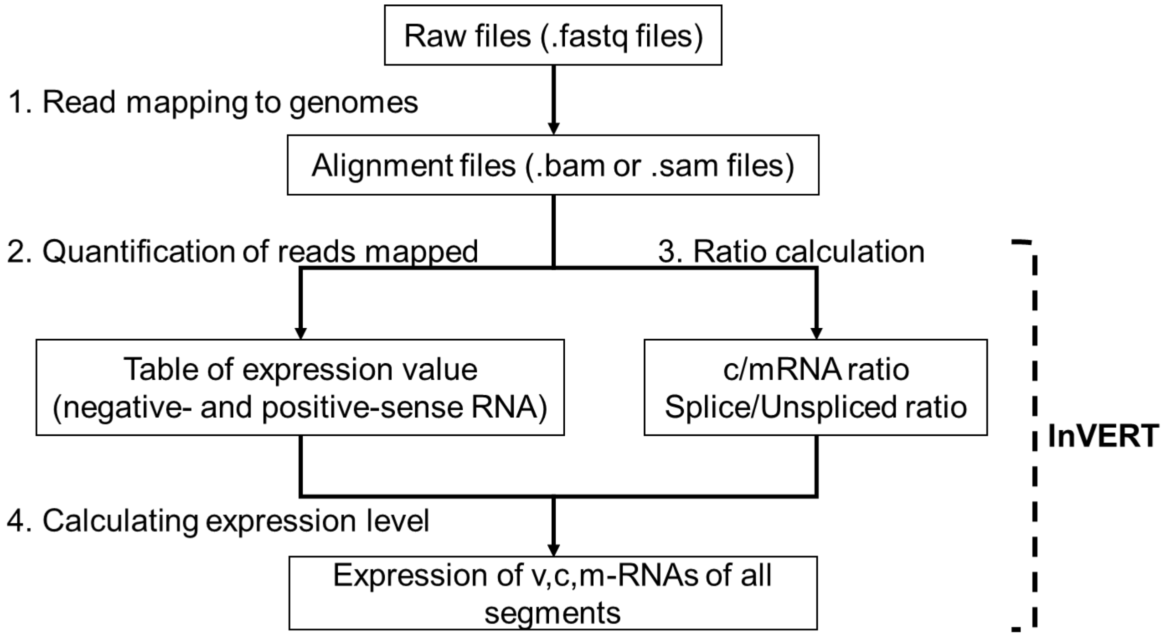

Figure S1. Data analysis procedure for analyzing IAV kinetics. Flow chart depicting each step of the data processing pipeline to determine IAV mRNA, cRNA and vRNA. Detailed descriptions of steps 1-4 can be found in the supplemental materials.

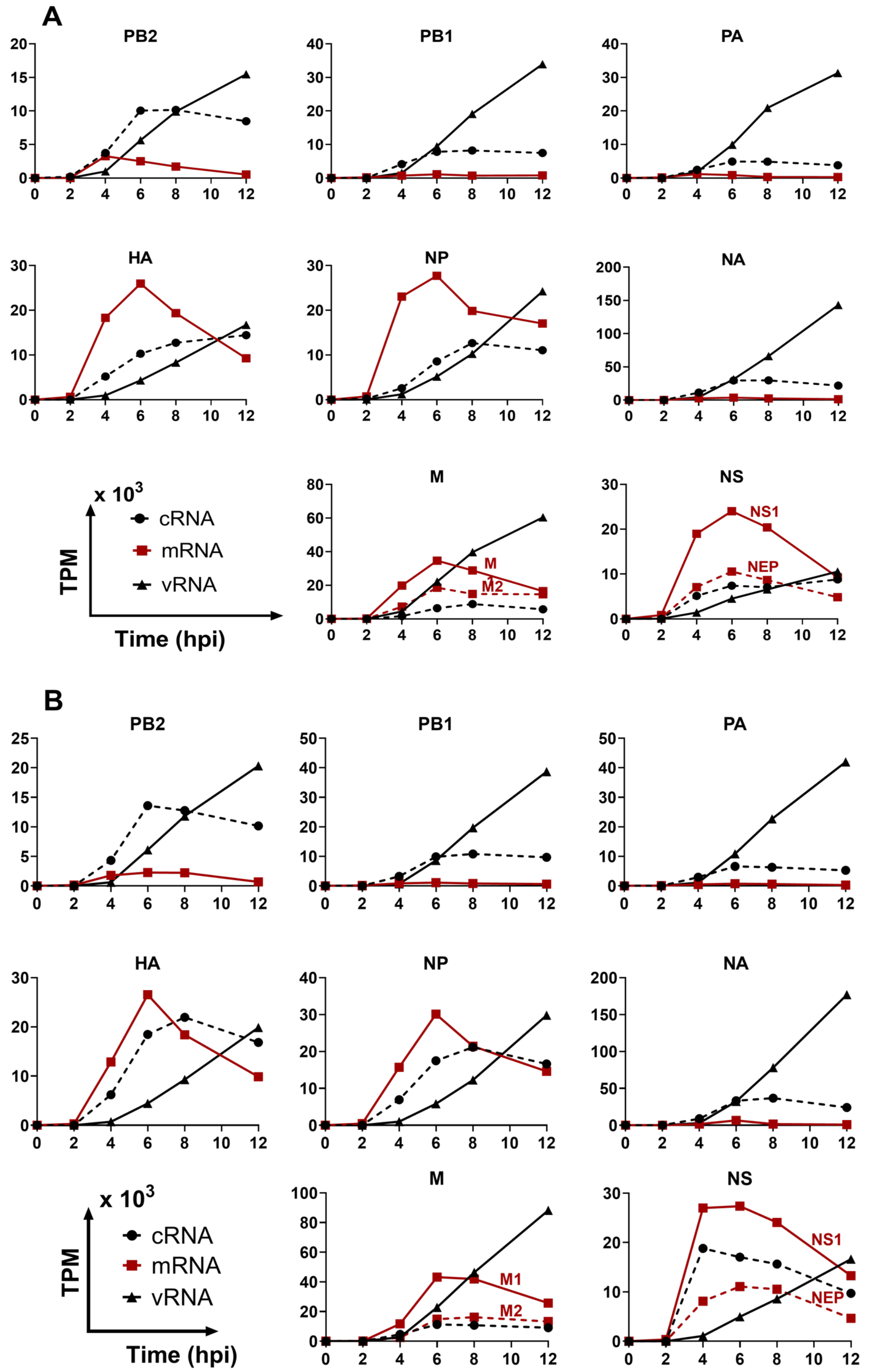

Figure S2. Kinetic profile of single-cycle infection of IAV PR/8/34 into MDCK. Each panel in this trellis graph shows the kinetics of vRNA (▲), cRNA (●), mRNA (■) of one viral segment along with time post-infection. Each graph (A) or (B) shows kinetic profile of one infection.

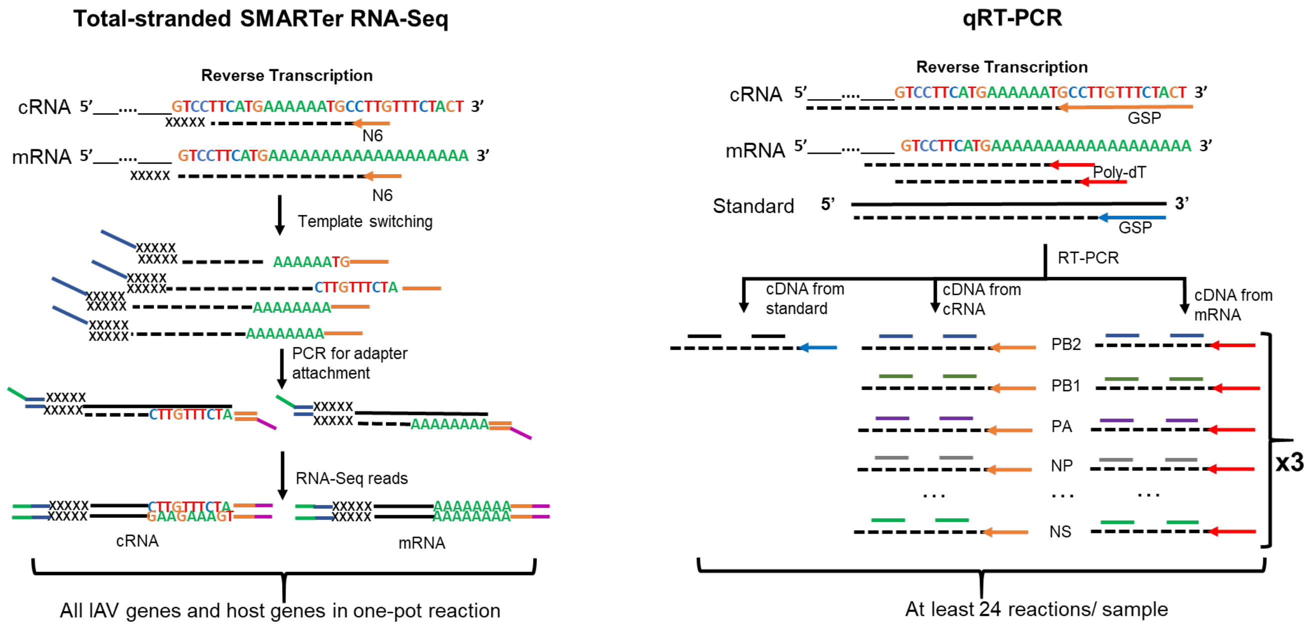

Figure S3. Comparison between total stranded RNA-Seq and qRT-PCR for quantifying virus replication kinetics. For the total-stranded RNA-Seq (left), cDNA is generated from total RNA using random hexamers (N6) as primers and the template switching facilitates the attachment of adapters for sequencing. After RNA-Seq, the differentiation of mRNA and cRNA was done using the analysis pipeline InVERT. For qRT-PCR (right), cDNA from each RNA species (cRNA or mRNA) was synthesized from reverse transcription with either gene-specific primer (GSP) for cRNA or Poly-dT primer for mRNA. cDNA from standard (either *in vitro* synthesized RNAs or housekeeping genes) also needs to be synthesized. The cDNA from each reverse transcription is the input of qPCR with GSPs for quantification. To quantify expression of 8 viral genes for all three RNA species, there need to be at least 24 qPCR reactions not including the standard and replicate for one sample.

Supplemental Methods

Step-by-step protocol for using InVERT to study influenza kintetics.

1. The raw files after sequencing (fastq files) after preprocessing (adapter trimming, QC) were mapped into both host and IAV genomes. The mapping of reads into host genome is optional and should not interfere with the quantification of IAV RNAs. Although a variety of mapping tools can be used in this step (bwa, Tophat, Bowtie, HISAT2, etc.), we used STAR as this RNA-Seq aligner allows accurate and ultrafast alignment of RNA-Seq reads into genomes. Refer to Box 1 for an example of using STAR for read alignment.

Note: Either pair-end or single-end sequencing can be used, as long as the read orientation is known.

Box 1. Parameters used for mapping RNA-Seq reads

```
STAR --runThreadN 16 --sjdbGTFfile $ANNOT  --sjdbOverhang 149 --outFilterType BySJout --
outFilterMultimapNmax 10 --alignSJoverhangMin 5 --alignSJDBoverhangMin 1 --outFilterMismatchNmax
999 --outFilterMismatchNoverReadLmax 0.04 --alignIntronMin 20 --alignIntronMax 1000000 --
alignMatesGapMax 1000000 --outFilterIntronMotifs RemoveNoncanonicalUnannotated --
outFileNamePrefix $OUT/ --outSAMtype BAM SortedByCoordinate --runMode alignReads --genomeDir
./index --readFilesIn $R1 $R2
```

$ANNOT directs to the annotation file, $OUT is the output name prefix, $R1 and $R2 are forward and reverse sequencing output files (fastq files), correspondingly. The output of STAR mapping by STARs would be alignment files (.bam files). The mapping statistics including mapping speed, uniquely mapped reads number/percentage, etc. were exported in the Log.final.out by STAR or reported by samtools [14] flagstat.

2. The reads mapped into the genome can be quantified by any isoform quantification tools (Cufflinks, HTSeq, featureCounts, RSEM, etc.) to obtain expression level of genes. The expression level of genes can be in any unit of choice (FPKM, RPKM, TPM, CPM, etc.). The output is a table of expression levels of all the genes.
Note: It is important to provide an annotation with both positive- and negative-strand RNA for virus genes. Providing the annotation for both positive- and negative-strand RNA allows quantification of the two RNA species. The example of annotation for segment PB2 and PA is shown below:

**EF467818.1**	**Genbank**	**CDS**	**2**	**2340**	**.**	**+**	**0**	**transcript_id "956536"; gene_id "956536"; gene_name "PB2";**
**EF467818.1**	**Genbank**	**CDS**	**1**	**2341**	**.**	**-**	**0**	**transcript_id "9565361"; gene_id "9565361"; gene_name "vPB2";**
**EF467820.1**	**Genbank**	**CDS**	**2**	**2232**	**.**	**+**	**0**	**transcript_id "956535"; gene_id "956535"; gene_name "PA";**
**EF467820.1**	**Genbank**	**CDS**	**1**	**2333**	**.**	**-**	**0**	**transcript_id "9565351"; gene_id "9565351"; gene_name "vPA";**

3. The calculation of cRNA:mRNA ratio is performed on positive-sense RNAs of IAV. The pipeline includes three main steps as below. The full script to perform Step 3.1-3.3 for IAV PR8 are posted on Github (https://github.umn.edu/phanx247/IAVKinetics)

3.1. Separation of reads mapped into positive-sense and negative-sense mapped reads. Refer to Box 2 for example of separating reads by their direction using samtools.

Box 2. Separation of reads into positive-sense or negative-sense reads by samtools

```
## FILE is the alignment file used as input

## Reverse strand

# 1. alignments of the second in pair if they map to the forward strand
samtools view -h -b -f 128 -F 16 $FILE >  ${FILE}_rev1.bam
# 2. alignments of the first in pair if they map to the reverse  strand
samtools view -h -b -f 80 $FILE > ${FILE}_rev2.bam
# Combine alignments that originate on the reverse strand.
samtools merge -f  ${FILE}_rev.bam  ${FILE}_rev1.bam ${FILE}_rev2.bam


## Positive strand

# 1. alignments of the second in pair if they map to the reverse strand
samtools view -h -b -f 144 $FILE >  ${FILE}_fwd1.bam
# 2. alignments of the first in pair if they map to the forward strand
samtools view -h -b -f 64 -F 16 $FILE >  ${FILE}_fwd2.bam
# Combine alignments that originate on the forward strand.
samtools merge -f  ${FILE}_fwd.bam  ${FILE}_fwd1.bam ${FILE}_fwd2.bam
```

$BAM is the alignment file from STAR mapping and is used as the input. The outputs are bam files for negative-sense and positive-sense RNAs (${BAM}_rev.bam and ${BAM}_fwd.bam, respectively).

3.2. Counting the number of reads mapped to cRNA and mRNA at the 3' end among all positive reads and calculating the fraction of cRNA and mRNA reads. Refer to Box 3 for example of this step for 2 gene segments - PB2 and PB1.

Box 3. An example of counting reads mapped into cRNAs and mRNAs and calculating the ratio of cRNA and mRNA for segment 1 and 2.

```
SAMPLES="${FILE}_fwd_sort.bam"
    mkdir ${FILE}_cmRNA
    cd ./${FILE}_cmRNA
# Filter out reads at the 3'-end that contains the 5As consensus sequence
    samtools view $SAMPLES EF467818.1:2325-2341  -o PB2.bam
    samtools view $SAMPLES EF467819.1:1001-1016  -o PB1.bam

# Count the number of reads that map to cRNAs
    cPB2=$(samtools view PB2.bam|awk 'BEGIN {FS="\t"}; {print $10}'|grep "AAAAACG"|wc -l)
    cPB1=$(samtools view PB1.bam|awk 'BEGIN {FS="\t"}; {print $10}'|grep "AAAAAATG"|wc -l)

# Count the number of reads that map to mRNAs
    mPB2=$(samtools view PB2.bam|awk 'BEGIN {FS="\t"}; {print $10}'|grep "AAAAAAA"|wc -l)
    mPB1=$(samtools view PB1.bam|awk 'BEGIN {FS="\t"}; {print $10}'|grep "AAAAAAAA"|wc -l)

# Print out result of the count
    echo "PB2" $mPB2 $cPB2 >> cmRatio.txt
    echo "PB1" $mPB1 $cPB1 >> cmRatio.txt

# Calculating the ratio of mRNA/(cRNA+mRNA) and cRNA/(cRNA+mRNA), respectively
    awk '{$4=$2/($2+$3); $5=$3/($2+$3); print}' cmRatio.txt
```

${FILE}_fwd_sort.bam is the alignment file (bam file) of the forward RNAs that is used as the input. The output is a table that contain 5 columns, including gene name, count of reads at 3' end that maps to mRNA, count of reads at 3' end that maps to cRNA, fraction of mRNA reads (mRNA/(cRNA+mRNA)), fraction of cRNA reads (cRNA/(cRNA+mRNA)).

EF467818.1:2325-2341 is the viral chromosomal coordination that is used to extract reads that contains the 5As, in which EF467818.1 is segment ID for PB2, and 2325-2341 is the coordination. The segment ID and the coordination changes upon the virus strain.

3.3. Calculating the ratio of spliced: unspliced isoform for alternative splicing genes (M and NS)

For alternatively spliced genes (M and NS), mRNAs further differentiate into unspliced and spliced mRNAs. The algorithm to determine mRNA expression of the alternatively spliced isoforms of these two genes are explained as in Box 4.

Box 4. Algorithm to determine mRNA expression of the alternatively spliced isoforms M1 and M2:

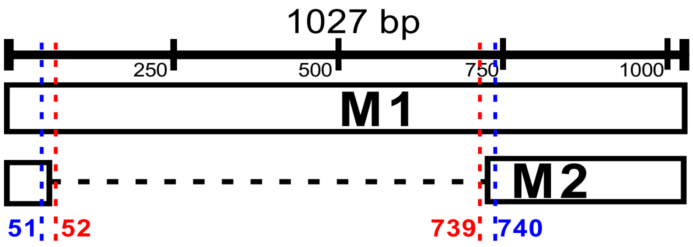

This diagram illustrates the splice sites of mRNA of segment M. The coverage of the closest base of exon at the junction (51 or 740, highlighted as dotted blue line in the diagram) consists of positive-sense cRNA (cM) and total mRNA (mM) which is further consists of unspliced M1 and spliced M2. The coverage of the closest base of intron at the junction (52 or 739, highlighted as dotted red line in the diagram) is contributed by only cM and M1.

Denote Transcript Per Million (TPM) by T and the read count by *c*, then:

$$c(M2) = \frac{1}{2}((c(51) - c(52)) + (c(740) - c(739))$$

$$c(mM) = \frac{1}{2}(c(51) + c(740)).\frac{mM}{cM+mM}$$

The ratio $\frac{mM}{cM+mM}$ is determined from step 3.2. Read count at base 51 and 52 (*c(51)* and *c(52)*, respectively) were determined by samtools as below:

```
## At the 5' splice site
    #Number of reads from both unspliced and spliced transcript
   samtools depth -m, -d 0 -r EF467824.1:51-51 $SAMPLES
    #Number of reads from unspliced transcript
   samtools depth -m, -d 0 -r EF467824.1:52-52 $SAMPLES
```

The output is a table of read count at bases of the splice sites that can be used to calculate the fraction of M2 in total mRNA (*f*):

$$f = \frac{c(M2)}{c(mM)} = \frac{c(M2)}{c(M1)+c(M2)}$$

4. Expression value of cRNA and mRNA can be calculated from the table of expression getting from step 2 and the output of step 3.2. In the case expression level is TPM:

$$TPM\ (mRNA) = \frac{mRNA}{cRNA + mRNA} \times TPM$$

$$TPM\ (cRNA) = \frac{cRNA}{cRNA + mRNA} \times TPM$$

The expression levels of alternative spliced isoforms of M and NS can be determined from the expression level of mRNA and the fraction M2 from total mRNA (calculated in step 3.3). The TPM values are adjusted to account for the size difference between unspliced and spliced transcripts, because spliced transcripts are shorter but Cufflinks and InVERT calculated TPM for only the full-length transcripts. For example, the expression level of M1 and M2 can be calculated by:

$$TPM(M2) = TPM(mM) \times \frac{1027}{\frac{c(52)}{c(51)} \times 1027 + \frac{c(M2)}{c(51)} \times 338} \times f$$

$$T(M1) = TPM(mM) \times \frac{1027}{\frac{c(52)}{c(51)} \times 1027 + \frac{c(M2)}{c(51)} \times 338} \times (1 - f)$$
